# Case Report: Clinical Features of a COVID-19 Patient With Cirrhosis

**DOI:** 10.3389/fmed.2021.678227

**Published:** 2021-11-26

**Authors:** Jian Zhou, Dixuan Jiang, Wanchun Wang, Kang Huang, Fang Zheng, Yuanlin Xie, Zhiguo Zhou, Jingjing Sun

**Affiliations:** ^1^Department of Orthopedics, The Second Xiangya Hospital, Central South University, Changsha, China; ^2^Department of Respiratory Medicine, The First Hospital of Changsha City, Changsha, China; ^3^Department of Anesthesiology, Second Affiliated Hospital, School of Medicine, Zhejiang University, Hangzhou, China

**Keywords:** COVID-19, cirrhosis, SARS-CoV-2, treatment, cured patient

## Abstract

Coronavirus disease 2019 (COVID-19) was first reported in Wuhan, Hubei Province, China in December 2019. At present, COVID-19 has emerged as a global pandemic. The clinical features of this disease are not fully understood, especially the interaction of COVID-19 and preexisting comorbidities and how these together further impair the immune system. In this case study, we report a COVID-19 patient with cirrhosis. A 73-year-old woman with cirrhosis reported a fever and cough on February 6, 2020. CT of the chest indicated an infection in her bilateral lungs. She tested positive for severe acute respiratory syndrome coronavirus 2 (SARS-CoV-2) infection. The woman was treated with lopinavir and ritonavir tablets and interferon alpha-2b injection, but there was no obvious effect. Although this patient was basically asymptomatic after 2 days in the hospital, the inflammation of the bilateral lungs was slow to subside as shown in CT of the chest. In addition, the white blood cell count (WBC), absolute neutrophil count, and absolute lymphocyte count remained decreased and the result of real-time reverse transcription polymerase chain reaction (PCR) (rRT-PCR) assay was still positive for SARS-CoV-2 on hospital day 28. After infusion of plasma from a recovered COVID-19 patient four times, the patient tested negative for SARS-CoV-2. She was discharged on March 13, 2020. This patient tested negative for SARS-CoV-2 after infusion of plasma from a recovered COVID-19 patient four times. Cirrhosis could impair the homeostatic role of the liver in the systemic immune response, which may affect the removal of SARS-CoV-2. This could lead to a diminished therapeutic effect of COVID-19. Thus, clinicians should pay more attention to COVID-19 patients with cirrhosis.

## Introduction

At present, many studies have indicated the epidemiological and clinical characteristics of coronavirus disease 2019 (COVID-19) (1–4). However, there are many diseases that may affect the immune system, such as AIDS, cirrhosis, and advanced malignant tumors, which may affect the removal of severe acute respiratory syndrome coronavirus 2 (SARS-CoV-2), further affecting the treatment of COVID-19 patients. A nationwide analysis in China analyzed the major strategies for patients with cancer in this COVID-19 crisis (5). The process of advanced cirrhosis is complicated with cirrhosis-associated immune dysfunction. Cirrhosis has the potential to injure the homeostatic role of the liver in the immune system (6, 7). In this case study, we report a case of a COVID-19 patient with cirrhosis. We describe the symptoms, diagnosis, treatment, and management of this patient, which may provide more information for the treatment of COVID-19 patients with cirrhosis.

## Case Report

On February 11, 2020, a 73-year-old woman came to the Fever Clinic of the First Hospital of Changsha, China. Ten minutes later, she was taken to the examination room and evaluated by a clinic doctor. The chief complaint of the patient was a fever—her body temperature peaked at 39°C—with cough, expectoration, shortness of breath, and general weakness that started prior 5 days. She developed mild diarrhea (3–4 stools/day) 2 days prior to coming to the hospital. Her daughter was diagnosed with COVID-19. Given her symptoms and recent close contact with a COVID-19-positive patient, she decided to go to a healthcare provider. The patient had a history of cirrhosis and type 2 diabetes, but no history of smoking or drinking. Physical examination indicated a body temperature of 38.8°C, a pulse of 100 beats/min, a respiratory rate of 22 breaths/min, an oxygen saturation of 85%, and bowel sounds at four times/min. She presented with a characteristic feature of chronic liver disease, hepatic facies, and liver palms, but no spider nevus. In addition, she had thick breathing sounds on both sides of the lungs and audible wet murmurs in both the lungs. The abdomen of the patient was soft and had no lumps. No pain was found in the liver without mobile dullness.

**Figure 1:**
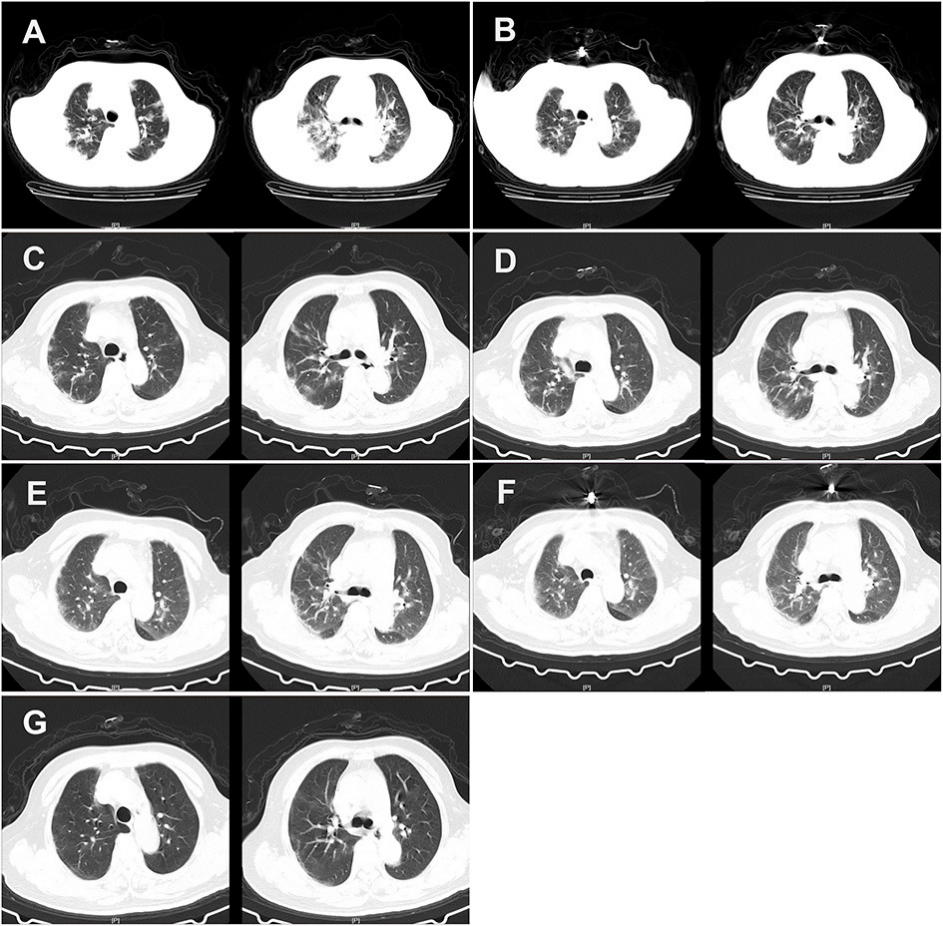
CT of the chest of the patient. **(A)** CT of the chest was obtained on February 12, 2020 (hospital day 2, illness day 6). The major morphogenesis of her bilateral lungs took on increased bronchovascular shadows and multiple patchy and maculas shadows, with cord-like ground-glass opacity (GGO) in the middle and lower regions of the lung. CT scan of the chest also showed increased lung markings. The texture of the trachea and blood vessels in both the lungs became thicker. **(B)** CT of the chest was obtained on February 16, 2020 (hospital day 6, illness day 10). The patchy lesions and maculas in both the lungs were partially absorbed. Increased lung markings were observed in the bilateral lungs. **(C)** CT of the chest was obtained on February 20, 2020 (hospital day 10, illness day 14). Decreased density of the patchy lesions in both the lungs was observed. The texture of the trachea and blood vessels in both the lungs became thicker. **(D)** CT of the chest was obtained on February 24, 2020 (hospital day 14, illness day 18). The pulmonary lesions remained unchanged. **(E)** CT of the chest was obtained on February 28, 2020 (hospital day 18, illness day 22). There was no obvious change in the patchy lesions in both the lungs. GGO was slightly increased. **(F)** CT of the chest was obtained on March 3, 2020 (hospital day 22, illness day 26). The major lesions of the bilateral lungs were not absorbed. **(G)** CT of the chest was obtained on March 10, 2020 (hospital day 29, illness day 33). The multiple patchy and maculas shadows of the bilateral lungs were further absorbed and the bronchovascular shadows were reduced.

Considering the possibility of SARS-COV-2 infection, we performed a chest CT examination and found bilateral pneumonia (Figure 1). The results of a nucleic acid amplification test (NAAT) for influenza A and B were negative. Her blood tests demonstrated simultaneous reduction of the ternary systems (red blood cells: 2.83 × 10^12^ cells/l; peripheral blood hemoglobin: 83 g/l; white blood cells: 0.78 × 10^9^ cells/l; lymphocytes: 0.11 × 10^9^ cells/l; lym%: 14.5%; platelets: 41 × 10^9^ cells/l) and an elevated percentage of neutrophils (0.65 × 10^9^/L; n%: 82.8%), C-reactive protein (62.5 mg/l), and erythrocyte sedimentation rate (129 mm/h) (Table 1). In view of the close contact history and clinical examination results of the patient, we carried out COVID-19 test for the patient. Specimens were collected following the Chinese Center for Disease Control and Prevention (CCDC) guidance. The results showed that she tested positive for SARS-COV-2. Therefore, she was admitted to the isolation ward for further treatment.

**Table 1:** Clinical laboratory results.

**Measure**	**Reference range**	**F11/H1**	**F12/H2**	**F15/H5**	**F17/H7**	**F19/H9**	**F20/H10**	**F22/H12**	**F25/H15**	**F28/H18**	**M2/H21**	**M6/H25**	**M10/H29**
qRT-PCR	N	P	-	P	-	-	P	-	P	N	P	P	N^T^
White cell count (10^9^/L)	4–10	0.78^*^	4.09	1.65^*^	4.47	3.40^*^	2.52^*^	1.86^*^	0.99^*^	1.01^*^	1.01^*^	0.78^*^	0.94^*^
Red cell count (10^12^/L)	3.5–5.5	2.83^*^	3.05^*^	3.02^*^	3.00^*^	3.54	3.19^*^	2.93^*^	2.68^*^	2.41^*^	2.38^*^	2.68^*^	2.78^*^
Absolute neutrophil count (10^9^/L)	2-7	0.65^*^	3.64^*^	1.36^*^	4.13	3.01	2.04	1.58^*^	0.73^*^	0.66^*^	0.66^*^	0.41^*^	0.56^*^
Absolute lymphocyte count (10^9^/L)	0.8–4	0.11^*^	0.22^*^	0.19^*^	0.13^*^	0.16^*^	0.29^*^	0.14^*^	0.17^*^	0.20^*^	0.23^*^	0.31^*^	0.28^*^
Monocyte (10^9^/L)	0.12–1.2	0.02^*^	0.18	0.09^*^	0.20	0.13	0.18	0.14	0.09^*^	0.07^*^	0.10^*^	0.06^*^	0.09^*^
Basophil (10^9^/L)	0.00-0.10	0.00	0.02	0.01	0.01	0.01	0.00	0.00	0.00	0.00	0.01	0.00	0.01
Eosinophil (10^9^/L)	0.02–0.5	0.00^*^	0.03	0.00^*^	0.00^*^	0.09	0.01^*^	0.00^*^	0.00^*^	0.00^*^	0.01^*^	0.00^*^	0.00^*^
Procalcitonin (ng/ml)	0–0.05	<0.05	<0.05	<0.05	<0.05	<0.05	<0.05	<0.05	<0.05	<0.05	<0.05	<0.05	<0.05
Platelets transfusion (10^9^/L)	100–300	41^*^	47^*^	44^*^	46^*^	60^*^	55^*^	45^*^	40^*^	31^*^	26^*^	35^*^	50^*^
Erythrocyte sedimentation rate (mm/h)	0-15	129^#^	-	-	103^#^	-	-	81^#^	82^#^	83^#^	100^#^	75^#^	71^#^
C-reactive protein (mg/L)	0–8	62.50^#^	-	9.50^#^	-	3.40	2.90	3.90	2.70	1.60	2.10	0.80	1.10
Albumin (g/L)	35-55	26.20^*^	-	32.90^*^	-	29.30^*^	31.90^*^	30.70^*^	31.30^*^	32.60^*^	32.90^*^	34.1^*^	36.7
PaO_2_ (mmHg)	80–100	62^*^	89	83.3	66.4^*^	102	63^*^	85.2	66.3^*^	-	70^*^	83	87
PaO_2_/FiO_2_ (mmHg)	400–500	124^*^	178^*^	208^*^	201^*^	309^*^	300^*^	293^*^	315^*^	-	333^*^	321^*^	338^*^
Alanine aminotransferase (U/L)	0-42	8.20	-	14.50	-	21.70	35.80	27.70	23.10	18.10	12.30	10.6	12.7
Aspartate aminotransferase (U/L)	0–37	31.10	-	19.50	-	17.00	40.30	28.40	29.10	25.40	20.30	26.2	24.3
Total bilirubin (μmol/L)	3.4–20.5	14.20	-	26.70^#^	-	12.20	14.90	22.70^#^	13.50	11.20	12.00	11.7	10.9
D-Dimer (μg/mL)	0–1	0.36	-	6.45^#^	5.87^#^	-	10.70^#^	8.29^#^	6.43^#^	5.46^#^	6.77^#^	7.24^#^	5.03^#^
Prothrombin time (s)	0–15	13.8	-	14.2	-	16.2^#^	13.5	12.3	11.7	-	15.9^#^	15.7^#^	12.9
International normalized ratio	0.92–1.38	1.27	-	1.31	-	1.49^#^	1.25	1.14	1.08	-	1.47^#^	1.45^#^	1.19
Fibrinogen (g/L)	2.0–4.0	2.59	-	1.63^*^	-	0.96^*^	1.19^*^	2.14	2.21	-	1.97^*^	1.76^*^	1.89^*^
Activated partial thromboplastin time (s)	26.2–46.0	32.4	-	25.1^*^	-	22.7^*^	23.8^*^	28.0	31.6	-	34.3	35.1	35.4
Thrombin time (s)	8–15	15.3^#^	-	14.4	-	26.1^#^	20.7^#^	17.7^#^	15.6^#^	-	19.0^#^	17.6^#^	16.4^#^

* *The value in the patient was below normal*.

# *The value in the patient was above normal*.

T *Tested negative for three times (M10, M11, and M12) by qRT-PCR*.

*F, February; M, March; H, hospital day; qRT-PCT, quantificational Real-Time Polymerase Chain Reaction*.

On day 1 of the hospital stay (illness day 5), the patient was administered lopinavir and ritonavir tablets (2 pills BID peros), which were recommended by *the Diagnosis and Treatment of Pneumonitis with COVID-19 Infection* (DTPI) published by the National Health Commission of the People's Republic of China (PRC) and interferon alpha-2b injection (5 million IU added into 2 ml of sterile water, inhalation BID). To inhibit inflammation in the lungs, she was treated with methylprednisolone sodium succinate (40 mg QD, intravenously). Yellow-green expectoration predicted the presence of a bacterial infection and, as such, moxifloxacin hydrochloride and sodium chloride injection (0.4 g QD) were given intravenously to the patient as treatment. Moreover, other supportive treatments included human immunoglobulin (10 g QD, intravenously) for improving immunity, *Bifidobacterium lactobacillus* trifecta orally for regulating the intestinal flora, recombinant human granulocyte colony-stimulating factor for promoting cell proliferation, and ampeptide elemente tablets for promoting the formation of platelets.

On day 2 of the hospital stay (illness day 6), she was asymptomatic apart from a cough, expectoration, chest tightness, and shortness of breath. Additionally, her temperature dropped to 36.9°C, but she reported that diarrhea still existed, approximately four times/day (Table 2). CT scans showed that the patchy infiltration was scattered as a small range of ground-glass opacity effusion and strip lesions in the bilateral lungs, which was similar to day 1 in the hospital (Figure 1). Otherwise, the laboratory results reflected that there was still a reduction in the tertiary system and hypoproteinemia due to liver dysfunction. Human serum albumin (50 ml BID) was then given intravenously. To prevent of episodes of hepatic encephalopathy, which is a chronically debilitating complication of hepatic cirrhosis, lactulose was added to the therapeutic regimen of the patient and nutritious meals were supplied to improve her anemia. The CCDC repeatedly confirmed that the oropharyngeal swabs of this patient tested positive for SARS-CoV-2 by real-time reverse transcription PCR (rRT-PCR) assay.

**Table 2:** Body temperatures and symptoms from February 6 to March 13, 2020.

**Date**	**F6–8**	**F9–10**	**F11**	**F12**	**F13**	**F14**	**F15**	**F16**	**F17**	**F18**	**F19**	**F20**	**F21**	**F22**	**F23**	**F24**	**F25**	**F26**	**F27**	**F28**	**F29–M13**
Illness day	Home	Home	5	6	7	8	9	10	11	12	13	14	15	16	17	18	19	20	21	22	23–36
Hospital day			1	2	3	4	5	6	7	8	9	10	11	12	13	14	15	16	17	18	19–32
Fever (°C)	Fever	Fever	38.8	36.9	36.0	36.0	36.6	36.0	36.1	36.2	36.4	36.4	36.4	37.7	37.0	37.0	36.7	36.7	36.0	36.0	36.1–36.9
Cough	√	√	√								√	√	√	√	√	√	√	√	√	√	
Shortness of breath	√	√	√								√										
Chest distress																					
Fatigue	√	√	√																		
Headache																					
Sore throat																					
Chest pain																					
Diarrhea		√	√	√	√	√	√	√	√	√	√			√	√	√	√				

*F, February; M, March*.

On day 3 of the hospital stay (illness day 7), the patient reported she felt better. Her pulse oxygen saturation increased significantly, up to 100%, at an oxygen flow rate of 2 l/min. Since that she still had diarrhea symptoms and lactulose was stopped to avoid the occurrence of imbalance of water and electrolytes. On day 4 of her hospital stay (illness day 8), a gastroenterologist was contacted to evaluate the persistent diarrhea of the patient. According to the suggestion of the gastroenterologist, the patient was treated with pantoprazole enteric-coated tablets (40 mg QD orally) for acid suppression. In addition, reduced glutathione (0.6 g QD) was given intravenously to protect her liver from subsequent damage.

On days 5–10 of the hospital day (illness days 9–14), the patient reported that her diarrhea improved to a degree and her clinical condition improved with supportive care. On hospital day 6 of the hospital stay, CT scans showed that the partial patchy lesions in the bilateral lungs were absorbed compared with the CT images obtained previously (Figure 1). Given the clinical presentation of the patient, treatment with human serum albumin was stopped on day 6 of the hospital stay. Lopinavir and ritonavir tablets, methylprednisolone sodium succinate, moxifloxacin, ampeptide elemente tablets, pantoprazole enteric-coated tablets, and human immunoglobulin were stopped on day 8 of the hospital stay of the patient (Table 3). However, the clinical course of the patient continued to deteriorate in terms of her respiratory symptoms, who typically presented with a cough and shortness of breath. Thymosin (0.1 g QD) and plasma (200 ml) from recovered COVID-19 patients plasma were then given intravenously to boost the immunity of the patient. On day 9 of the hospital stay (illness day 13), the C-reactive protein of this patient dropped to 3.4 mg/l. Nevertheless, CT scans of the chest indicated that the symptoms of the bilateral lungs of the patient did not improve on day 10 of the hospital stay (Figure 1). Moreover, the oropharyngeal swabs of this patient retested positive. Therefore, chloroquine phosphate (0.5 g BID) was administered orally instead. Additionally, the treatments did not improve the level of blood cells because of liver dysfunction and hypersplenism caused by cirrhosis.

**Table 3:** Order sheet of the physician.

**Drug**	**Date**	**Hospital day**	**Dose**	**Usage**
Lopinavir and ritonavir tablets	F11–F18	H1–H8	2 pills BID	po
Interferon alfa-2b injection	F11–F25	H1–H15	5 million IU BID	inh
Methylprednisolone sodium succinate	F11–F18	H1–H8	40 mg QD	ivgtt
Bifidobacterium lactobacillus trifecta	F11–F11	H1–H1	2 g BID	po
Human immunoglobulin	F11–F18	H1–H8	10 g QD	ivgtt
Ampeptide elemente tablets	F11–F18	H1–H8	0.4 g TID	po
Human serum albumin	F12–F16	H2–H6	50 ml BID	ivgtt
Pantoprazole enteric-coated tablets	F14–F18	H4–H8	40 mg QD	po
Reduced glutathione for injection	F14–F29	H4–H19	0.6 g QD	ivgtt
Moxifloxacin hydrochloride and sodium chloride injection	F15–F18	H5–H8	0.4 g QD	ivgtt
Thymosin	F19–F19	H9–H9	0.1 g QD	ivgtt
Chloroquine	F20–F27	H10–H17	0.5 g BID	po
Montmorillonite powder	F25–F28	H15–H18	3 g QD	po
loperamide hydrochloride	F26–F28	H16–H18	2 mg QD	po

*F, February; H, hospital day; ivgtt, intravenously guttae; po, per os; inh, inhalation*.

On days 11–18 of the hospital stay (illness days 15–22), she was in good clinical condition, except for a persistent cough and intermittent diarrhea. In order to further alleviate the diarrhea of the patient, montmorillonite powder (3 g QD) and loperamide hydrochloride (2 mg QD) were administrated orally. Moreover, interferon alpha-2b injections were stopped due to its limited effect on the clearance of the virus and the plasma from recovered COVID-19 patients was infused again on day 15 of the hospital stay (illness day 19). As the diarrhea of the patient improved, antidiarrheal drugs were discontinued on day 18 of the hospital stay (illness day 22).

On days 19–29 of the hospital stay (illness days 23–33), the vital signs of the patient were largely stable. The patient reported that her cough and diarrhea had abated and her clinical condition improved. Given these good clinical conditions, a reduction in glutathione injections was initiated on day 19 of her hospital stay. However, since the oropharyngeal swabs of this patient tested positive again, she was treated with plasma from a recovered COVID-19 patient for the third time. On day 29 of the hospital stay (illness day 33), CT scans showed that the patchy lesions in the bilateral lungs of the patient had absorbed compared with the CT images obtained previously (Figure 1). On the same day, the patient tested negative for COVID-19 infection (Table 1). On day 30 of the hospital stay (illness day 34), the patient was once again treated with the plasma from a recovered COVID-19 patient in order to ensure that the virus was completely cleared. On days 30–31 of the hospital stay, the patient tested negative for COVID-19 by an rRT-PCR assay for two times. She was discharged on March 13, 2020 (day 32 of the hospital stay, illness day 36).

## Discussion

Cirrhosis affects the cellular and humoral immune response of the entire body and the immune system of the liver (6, 8). The proportion of CD4^+^/CD8^+^ cells in the liver of patients with cirrhosis decreases and the distribution of lymphocytes varies within different lesions. CD8^+^ cells predominate in the necrotic area, while CD4^+^ cells increase in the manifold area. T-helper type 1 (Th1) cells dominate during the early stages of cirrhosis and then gradually drift toward Th2 cells. In order to understand the impact of cirrhosis on the treatment of COVID-19, we report the symptoms, diagnosis, treatment, and management of a COVID-19 patient with cirrhosis.

In this case study, the patient tested positive for SARS-CoV-2, which was supported by CT scan of the chest and she was admitted to the isolation ward at the First Hospital of Changsha City, China. Lopinavir and ritonavir tablets combined with interferon alpha-2b injections were given to her on her first day in the hospital. Though she was basically asymptomatic on day 2 of her hospital stay and her body temperature also returned to a normal range, the inflammation of her bilateral lungs was difficult to subside, suggesting that clinicians should be aware of COVID-19 patients with diseases affecting the immune system. These patients may show mild or even no symptoms, while the inflammation of lungs may be progressing. Therefore, if a person with basic diseases that impair the immune system was exposed to confirmed COVID-19 cases, they should immediately come to the hospital even if they have no symptoms. Also, doctors need to be aware of the progression of inflammation in the lungs.

Previous reports showed that COVID-19 patients with cirrhosis had lower albumin than patients with COVID-19 (9), which was consistent with the results of this case study. Moreover, Qi et al. discovered that leukopenia, lymphopenia, and thrombocytopenia occurred in COVID-19 patients with cirrhosis (10), which were similar to the results we obtained. Additionally, increasing evidence indicated that patients with COVID-19 exhibited a hypercoagulability in the lung (11). In this case study, the D-dimers of COVID-19 patient with cirrhosis were elevated, suggesting hypercoagulability of the patient. The liver synthesizes a variety of coagulation factors. When cirrhosis causes liver insufficiency, the production of coagulation factors is reduced, which leads to prolonged prothrombin time (PT), activated partial thromboplastin time (APTT) and thrombin time (TT), and a decrease of fibrinogen. Therefore, the PT, APTT, and TT of COVID-19 patient with cirrhosis were prolonged and fibrinogen was decreased, which was similar to the previous study (12). In addition, venous thromboembolism (VTE) including deep venous thrombosis (DVT) is common in cirrohsis patients. Additionally, the patient in this case study was treated with antiviral drugs, which had no obvious effect on her symptoms. Previous study indicated that 96% cirrhotic patients with confirmed SARS-CoV-2 infection needed hospitalization or prolonged an ongoing one (13). In this case study, we observed similar results. This COVID-19 patient with cirrhosis was hospitalized for 32 days. She was tested positive for COVID-19 on day 25 of her hospital stay. Moreover, the numbers of WBC and the absolute neutrophil count and absolute lymphocyte count remained reduced in this patient. The process of advanced cirrhosis is complicated with cirrhosis-associated immune dysfunction. Cirrhosis has the potential to injure the homeostatic role of the liver in the immune system, which may be associated with the process of COVID-19. Additionally, although the mortality of COVID-19 was mediated by pulmonary involvement, cirrhosis is assumed to be a high-risk factor for severe COVID-19 because of an altered gut-liver axis and inherent immune dysfunction. Cirrhosis can impair the cellular and humoral immune system of the entire body, which may impair the removal of SARS-CoV-2. Thus, physicians may need to monitor immune indicators in COVID-19-positive patients with comorbidities that impair the immune system.

The patient in this case study was administered the plasma (200 ml) from recovered COVID-19 patients four times. After the last administration of plasma on day 30 of the hospital stay, the patient tested negative for SARS-CoV-2 three consecutive times and then she was discharged on day 32 of her hospital stay. This suggested that the treatment for COVID-19 is passive immunotherapy. Cirrhosis can impair the homeostatic role of the liver in the systemic immune response; thus, passive immunotherapy, such as plasma administration from recovered COVID-19 patients, may be an option for treatment. However, this case study has a limitation that needs to be cautious. These findings have only been observed in one patient. Further multicenter with large sample studies are needed to perform to verify the results.

## Conclusion

This case study described the symptoms, diagnosis, treatment, and management of a COVID-19 patient with cirrhosis, emphasizing the need to pay attention to underlying diseases in COVID-19-positive patients. More information about this disease is still needed in order to successfully explore its clinical management.

## Data Availability Statement

The datasets used and/or analyzed during the current study are available from the corresponding author upon reasonable request.

## Ethics Statement

The studies involving human participants were reviewed and approved by The First Hospital of Changsha City Committee for Clinical Research. The patients/participants provided their written informed consent to participate in this study. Written informed consent was obtained from the individual(s) for the publication of any potentially identifiable images or data included in this article.

## Author Contributions

JZ and JS conceived and designed the study and also critically revised the manuscript. JZ and WW conducted the experiments and drafted the manuscript. DJ, KH, FZ, YX, and ZZ contributed to the revision of the manuscript. All authors have read and approved the final manuscript.

## Funding

This study was funded by the Innovative Major Emergency Project Funding against the New Coronavirus Pneumonia in Hunan Province (Grant Nos. 2020SK3014 and 2020SK3013), the Key Research & Developmental Program of Hunan Province (2022SK2047), Chinese Public Health Union (GWLM202039), Health and Family Planning Commission Fund Project in Hunan Province (Grant No. B2017209), Natural Science Foundation of Hunan Province (Grant No. 2018JJ2452), New Coronavirus Pneumonia Emergency Project of Changsha Science and Technology Bureau (Grant Nos. kq2001010 and kq2001008), the Mittal Innovation Project of Central South University (Grant No. GCX20190879Y) and the Fundamental Research Funds for the Central Universities of Central South University (Grant No. 2018zzts930). The study funders/sponsors had no role in the design and conduct of the study; collection, management, analysis, and interpretation of the data; preparation, review, or approval of the manuscript; and decision to submit the manuscript for publication.

## Conflict of Interest

The authors declare that the research was conducted in the absence of any commercial or financial relationships that could be construed as a potential conflict of interest.

## Publisher's Note

All claims expressed in this article are solely those of the authors and do not necessarily represent those of their affiliated organizations, or those of the publisher, the editors and the reviewers. Any product that may be evaluated in this article, or claim that may be made by its manufacturer, is not guaranteed or endorsed by the publisher.

## References

[1] Wan S Xiang Y Fang W Zheng Y Li B Hu Y. Clinical features and treatment of COVID-19 patients in northeast Chongqing. J Med Virol. (2020) 92:797–806. 10.1002/jmv.25783 32198776 PMC7228368

[2] Chen N Zhou M Dong X Qu J Gong F Han Y. Epidemiological and clinical characteristics of 99 cases of 2019 novel coronavirus pneumonia in Wuhan, China: a descriptive study. Lancet. (2020) 395:507–13. 10.1016/S0140-6736(20)30211-7 32007143 PMC7135076

[3] Huang C Wang Y Li X Ren L Zhao J Hu Y. Clinical features of patients infected with 2019 novel coronavirus in Wuhan, China. Lancet. (2020) 395:497–506. 10.1016/S0140-6736(20)30183-5 31986264 PMC7159299

[4] Holshue ML DeBolt C Lindquist S Lofy KH Wiesman J Bruce H. First Case of 2019 Novel Coronavirus in the United States. N Engl J Med. (2020) 382:929–36. 10.1056/NEJMoa2001191 32004427 PMC7092802

[5] Liang W Guan W Chen R Wang W Li J Xu K. Cancer patients in SARS-CoV-2 infection: a nationwide analysis in China. Lancet Oncol. (2020) 21:335–7. 10.1016/S1470-2045(20)30096-6 32066541 PMC7159000

[6] Albillos A Lario M Alvarez-Mon M. Cirrhosis-associated immune dysfunction: distinctive features and clinical relevance. J Hepatol. (2014) 61:1385–96. 10.1016/j.jhep.2014.08.010 25135860

[7] Sipeki N Antal-Szalmas P Lakatos PL Papp M. Immune dysfunction in cirrhosis. World J Gastroenterol. (2014) 20:2564–77. 10.3748/wjg.v20.i10.2564 24627592 PMC3949265

[8] Kreivenaite E Gedgaudas R Valantiene I Mickiene A Kupcinskas J. COVID-19 in a Patient with Liver Cirrhosis. J Gastrointestin Liver Dis. (2020) 29:263–6. 10.15403/jgld-2440 32530994

[9] Bajaj JS Garcia-Tsao G Biggins SW Kamath PS Wong F McGeorge S. Comparison of mortality risk in patients with cirrhosis and COVID-19 compared with patients with cirrhosis alone and COVID-19 alone: multicentre matched cohort. Gut. (2021) 70:531–6. 10.1136/gutjnl-2020-322118 32660964 PMC7371484

[10] Qi X Liu Y Wang J Fallowfield JA Wang J Li X. Clinical course and risk factors for mortality of COVID-19 patients with pre-existing cirrhosis: a multicentre cohort study. Gut. (2021) 70:433–6. 10.1136/gutjnl-2020-321666 32434831 PMC7815629

[11] Jiang M Mu J Shen Zhang H. COVID-19 With Preexisting Hypercoagulability Digestive Disease. Front Med (Lausanne). (2020) 7:587350. 10.3389/fmed.2020.587350 33521013 PMC7838325

[12] Blasi A von Meijenfeldt FA Adelmeijer J Calvo A Ibañez C Perdoma J. *In vitro* hypercoagulability and ongoing *in vivo* activation of coagulation and fibrinolysis in COVID-19 patients on anticoagulation. J Thromb Haemost. (2020) 18:2646–53. 10.1111/jth.15043 32762118 PMC7436627

[13] Iavarone M D'Ambrosio R Soria A Triolo M Pugliese N Poggio PD. High rates of 30-day mortality in patients with cirrhosis and COVID-19. J Hepatol. (2020) 73:1063–71. 10.1016/j.jhep.2020.06.001 32526252 PMC7280108

